# Comfort evaluation and physiological effects/autonomic nervous system response of inflatable deep pressure vest in reducing anxiety

**DOI:** 10.1016/j.heliyon.2024.e36065

**Published:** 2024-08-09

**Authors:** Mohamad Izzur Maula, Muhammad Imam Ammarullah, Hanifa Nur Fadhila, Ilham Yustar Afif, Hardian Hardian, Jamari Jamari, Tri Indah Winarni

**Affiliations:** a Department of Mechanical Engineering, Faculty of Engineering, Universitas Diponegoro, Semarang, 50275, Central Java, Indonesia; b Undip Biomechanics Engineering & Research Centre (UBM-ERC), Universitas Diponegoro, Semarang, 50275, Central Java, Indonesia; c Department of Manufacturing Engineering Technology, Akademi Inovasi Indonesia, Salatiga, 50721, Central Java, Indonesia; d Sustainable Energy and Bioengineering Research Centre, University of Liberia, Monrovia, 1000, Montserrado, Liberia; e Department of Anatomy, Faculty of Medicine, Universitas Diponegoro, Semarang, 50275, Central Java, Indonesia; f Department of Mechanical Engineering, Faculty of Engineering, Universitas Muhammadiyah Semarang, Semarang, 50273, Central Java, Indonesia; g Department of Physiology, Faculty of Medicine, Universitas Diponegoro, Semarang, 50275, Central Java, Indonesia; h Center for Biomedical Research (CEBIOR), Universitas Diponegoro, Semarang, 50275, Central Java, Indonesia

**Keywords:** Deep pressure therapy, Anxiety, Comfort test, Pulse rate

## Abstract

**Background:**

Deep pressure therapy treats anxiety by triggering physiological responses and promoting calmness. Moreover, measuring user comfort can improve product quality.

**Objective:**

To investigate the physiological effects and subjective comfort level of inflatable deep pressure vests to enhance their calming effect.

**Methods:**

Experimental research was conducted with a one-group pretest-posttest design for physiological effects using pulse oximetry for peripheral pulse rate and a one-shot case study for three subscale parameters that help evaluate comfort (pressure, touch, and mobility) using the Numerical Rating Scale (NRS). Deep pressure intervention using the Inflatable Vest was performed for three sessions, each lasting 5 min.

**Results:**

This study was conducted with 46 participants (24 males, 22 females) aged 17–20 (19.52 ± 0.78). Although pulse rate consistently decreased in all sessions, session 1 showed a significant decrease (*p* = 0.014*, *d* = 0.379), whereas sessions 2 (*p* = 0.274, *d* = 0.163) and 3 (*p* = 0.597, *d* = 0.078) demonstrated non-significant decreases with small effect sizes. The pressure comfort subscale showed that 87.0 %, 4.3 %, and 8.7 % of the participants, and the touch comfort subscale test revealed that 82.6 %, 8.7 %, and 73.9 % of the participants rated it as comfortable, very comfortable, and less comfortable, respectively. The mobility subscale test showed that 73.9 % of the participants rated no limitation, 17.4 % rated somewhat limited, and only 8.7 % rated limitation. Decreased pulse rate and pressure comfort were significantly positively correlated (*r* = 0.282**, *p* < 0.01), whereas touch pressure and mobility were not (*r* = 0.160, *p* > 0.05; and *r* = 0.121, *p* > 0.05, respectively). Decreased pulse rate was also positively correlated with the overall score for the three aspects (*r* = 0.201*, *p* < 0.05)

**Conclusions:**

A comfortable inflatable deep pressure vest provides a physiologically calming effect for therapeutic modalities.

## 1 Introduction

The sense of touch plays a crucial role in human development and daily life and contributes to cognitive and social brain development [1,2]. Touch sensitivity encompasses four modalities: pressure/vibration, pain, itching, and temperature, each activating specific receptors in response to a stimulus. Mechanoreceptors in the skin and joints respond to pressure/vibration by distorting cation channel proteins, leading to an influx of Na + ions and triggering action potentials in afferent nerves [3]. These sensory inputs convey discriminative information regarding the tactile stimuli to the central nervous system. Gentle touch and deep pressure, such as hugging, are detected by low-threshold mechanoreceptors known as C-tactile afferents (CTs) in hairy skin, which provide effective and pleasing touch properties [4].

Anxiety is marked by feelings of tension, worried thoughts, and physical changes, such as an increase in blood pressure [5]. In addition to feelings of high panic intensity, sufferers may experience physiological symptoms such as fatigue due to the influence of excessively active sympathetic nerve fibers [6], headaches, palpitations, and shortness of breath. Anxiety has received little attention and often remains undetected or is left untreated [7]. The Riset Kesehatan Dasar (Riskesdas) Indonesia report of 2018 showed the prevalence of anxiety and depression in a young population aged 15–24 years as 6.2 % [8].

Current technological advancements have spurred studies exploring the provision of pressure stimuli via vests. One study involved active Shape Memory Alloy (SMA) compression garments, aiming to deliver pressure stimuli, particularly on the trunk and proximal extremities [9]. While offering emotional benefits, such as calming effects, active SMA compression garments pose safety and operational challenges owing to the risk of user disturbance or harm from the expansion-shrinkage of metal alloy springs [10].

Conversely, inflatable vests have been reported to offer superior calming effects compared with other pressure distribution methods, such as weighted or stretched tools, with simpler technological applications [[11], [12], [13]]. These varying effects may stem from distinct tactile stimulation styles generated by the inflated, pulled, or stretched parts. Inflatable vests provide a unique tactile experience characterized by gentle pressure and enveloping sensations theorized to promote relaxation and reduce stress. Considering the importance of user comfort, as highlighted by the World Health Organization (WHO), it is crucial to measure convenience in new product development to enhance product quality [14]. Understanding the nuances of tactile stimulation and its impact on the user experience can guide the refinement of inflatable vest designs to optimize their effectiveness in promoting relaxation and well-being.

The physiological effects of inflatable vests have been extensively examined; however, a notable gap exists in the literature regarding perceived comfort and its correlation with these effects. Consequently, this research explores the effectiveness of tactile stimulation imparted by an inflatable vest to enhance its calming effects and assesses the comfort experienced during its usage. This investigation seeks to improve the viability of an inflatable vest as a more streamlined and compact tool for deep-pressure therapy, thereby improving its therapeutic efficacy.

In this study, we hypothesize that the use of an inflatable vest will significantly reduce physiological markers of stress, such as heart rate and cortisol levels, compared to baseline measurements. Furthermore, we anticipate that the inflatable vest will be rated as more comfortable and effective in providing calming effects compared to other pressure distribution methods. To explore these hypotheses, we aim to understand how the tactile stimulation from an inflatable vest impacts subjective reports of relaxation and well-being. Additionally, we seek to investigate the relationship between the perceived comfort of the inflatable vest and its physiological calming effects.

## 2 Materials and methods

### 2.1 Study design and participants

This study was conducted at the Undip Biomechanics Engineering and Research Centre (UBM-ERC) Laboratory, Universitas Diponegoro, Semarang, Indonesia. Experimental research was conducted using a one-shot case study design for the comfort level investigation and a one-group pretest-posttest design for the short-term physiological effect.

Table 1 shows the inclusion and exclusion criteria in this study for determining the eligibility of the participant’s selection.

**Table 1:** Inclusion and exclusion criteria.

Criteria	Descriptions
Inclusion	• Adolescents aged 17–20 • Body Mass Index (BMI) of 18.5–24.9 kg/m^2^
Exclusion	• In an anxious state according to the Hamilton Anxiety Rating Scale • Has a deformity of the vertebral column • Has injuries and/or neuropathic abnormalities in the compression area (truncus)

Participants were administered a deep-pressure inflatable vest intervention for 5 min in each session, conducted over three consecutive days, to avoid bias from residual effects. The influence of session order on the therapeutic effects was not investigated in this study [15].

This study investigated the short-term effects of the use of inflatable vest. Data collection was performed thrice to strengthen the consistency of the results obtained using the inflatable vest. The collective influence of three sessions was not analyzed; however, each session was analyzed individually.

### 2.2 Ethics

An explanation of the study procedure was provided to all participants, and those who agreed to participate by signing informed consent were included. The study procedures were approved by the Health Research Ethics Commission, Faculty of Medicine, Universitas Diponegoro, Semarang (No. 373/EC/KEPK/FK-UNDIP/IX/2021).

### 2.3 Instruments

#### 2.3.1 The inflatable vest

In this investigation, the Squease™ vest (London, UK) was employed, undergoing customization by integrating an electric pump. This modification was aimed at attaining an elevated level of pressure adjustability, thereby enhancing the overall tactile experience within the study.

#### 2.3.2 Pulse oximetry

Pulse oximetry was used as the physiological measurement tool because of its easy usability and rapid delivery of results. The peripheral pulse and heart rate have a very strong correlation and can be used as stress indicators [16,17].

#### 2.3.3 The Numerical Rating Scale (NRS)

A Numerical Rating Scale (NRS) was used to assess the subjective comfort level associated with wearing the inflatable deep pressure vest. It comprised three questions pertaining to pressure comfort (Q1), touch comfort (Q2), and mobility (Q3). The scores ranged from 0 to 10, with interpretations ranging from extremely uncomfortable at a score of 0 to very comfortable score of 10.

### 2.4 Experimental procedures

#### 2.4.1 Pre experimental phase

Participants willing to participate in the study were explained the sequence of experiments, followed by completion of the Hamilton Anxiety Rating Scale (HARS). Participants who met the inclusion criteria provided written consent. Participants were instructed to sit in a chair in a standard posture: sitting symmetrically with the midline on the chair surface, maintaining an upright back position without support, keeping their feet horizontal on the footrest, and maintaining the distance between the lower knee and the front edge of the seat so that the thigh-foot angle was 95°.

#### 2.4.2 Experimental phase

Peripheral pulse was measured before wearing the inflatable vest for the pretest. During the measurements, participants were asked not to move their fingers. The peripheral pulse rate was measured three times, and the mean was obtained. The inflatable vest was worn by the participants, assisted by a researcher. They were then asked to press the INFLATE button to start inflating the vest until they reached the desired level, according to Reynold’s study [18]. After 5 min, the air was released by pressing the RELEASE button. Peripheral pulse rate measurements were performed again as a posttest, and participants were then asked to complete the NRS assessment.

#### 2.4.3 Post experimental phase

**Fig. 1:**
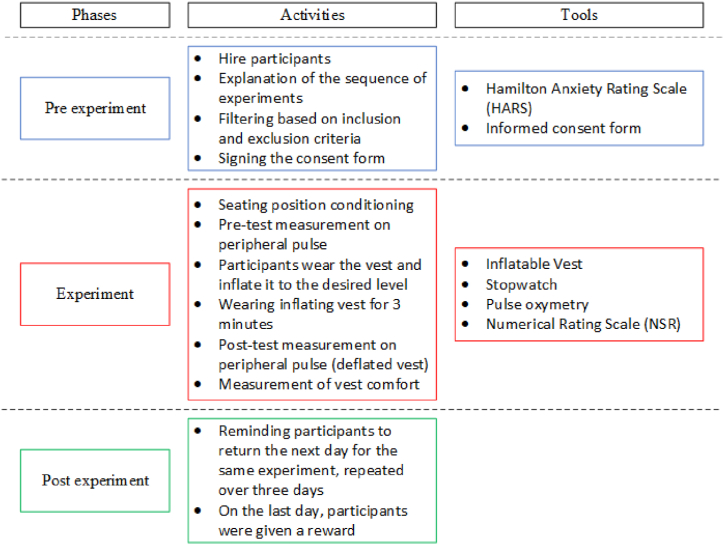
Phases, activities, and tools in the present study.

The entire procedure from start to finish was repeated thrice on different days (three consecutive days) to avoid residual effects. Participants were reminded to come the next day to undergo the same testing for three consecutive days. All phases, activities, and tools used in this study are shown in Fig. 1.

### 2.5 Statistical analysis

Shapiro–Wilk test was applied as a normality test, which indicated that the peripheral pulse data were normally distributed (*p* > 0.05). Subsequently, a paired *t*-test was conducted to analyze the differences between the pretest and posttest. The validation and reliability of the NRS questionnaire were also analyzed using Pearson product-moment correlation and Cronbach alpha, followed by categorical descriptive analysis to determine the distribution of the device comfort level. The Pearson correlation test was applied to analyze the correlation between pulse rate and comfort level. The significance level was set at a confidence interval (CI) of 95 % (*p* ≤ 0.05), and all statistical tests were conducted using IBM SPSS software.

## 3 Results

All 46 participants (24 males and 22 females, mean age 19.52 ± 0.78) who met the inclusion and exclusion criteria completed every phase of the experiment in this study. There were no significant differences in gender (male-female) observed in either peripheral pulse changes (*p* = 0.387) or comfort rate (*p* = 0.481).

### 3.1 Peripheral pulse activity

**Fig. 2:**
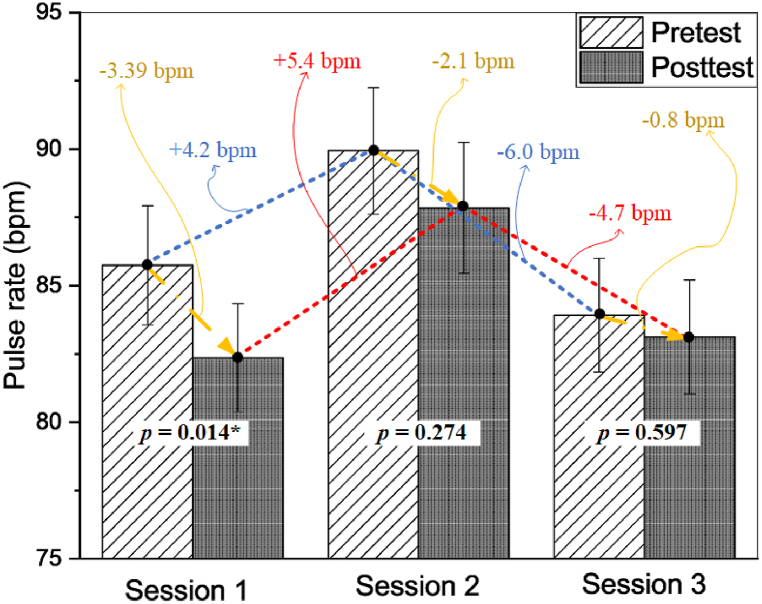
Changes in pretest-posttest peripheral pulse rate.

From Fig. 2, it is evident that there was a decrease in the pulse rate across all sessions, indicating a consistent calming effect. However, upon further evaluation, a significant calming effect was observed only in session one, with a small effect size (*p* = 0.014*, *d* = 0.379). Conversely, sessions two (*p* = 0.274, *d* = 0.163) and three (*p* = 0.597, *d* = 0.078) demonstrated decreases that were not significant and were accompanied by small effects. The changes in peripheral pulse rate and their effect sizes are detailed in Table 2.

**Table 2:** Changes in pulse rate.

Pulse	Pulse	Mean ± SD	Δ ± SD	95 % CI		*p*	Cohen *d*
				Lower	Upper		
Session 1	Pretest 1	85.75 ± 14.79	3.39 ± 8.96	0.731	6.052	0.014^a^	0.379
	Posttest 1	82.36 ± 13.46					
Session 2	Pretest 2	89.94 ± 15.72	2.09 ± 12.78	−1.708	5.882	0.274	0.163
	Posttest 2	87.85 ± 16.23					
Session 3	Pretest 3	83.91 ± 14.13	0.79 ± 10.08	−2.203	3.784	0.597	0.078
	Posttest 3	83.12 ± 14.16					

a *p* is significant at the 0.05 level (2-tailed).

### 3.2 Comfort rate

The Pearson correlation results, indicating high validity (Q1: *r* = 0.889**; Q2: *r* = 0.823**; Q3: *r* = 0.880**; Q1-Q3: *p* < 0.01), and the Cronbach alpha analysis demonstrates the acceptable reliability (α = 0.8) of the NRS for comfort measurement.

**Fig. 3:**
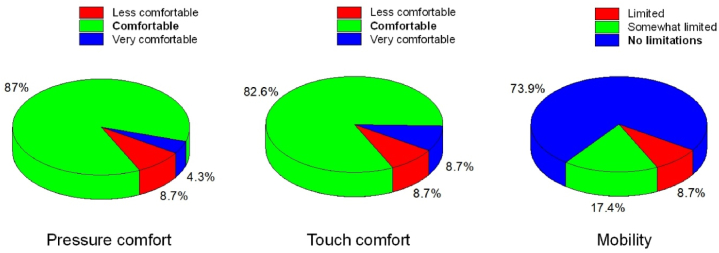
Comfort measurements.

The NRS scores were grouped into three categories: less comfortable (0–3), comfortable (4–7), and very comfortable (8–10); for pressure and touch comfort, limited (0–3), somewhat limited (4–7), and no limitations (8–10) for the mobility. As shown in Fig. 3, most respondents considered the inflatable vest to have comfortable pressure (87.0 %) and touch (82.6 %) and responded that they did not limit their mobility (73.9 %).

### 3.3 Correlation of pulse rate on comfort level

The results of the Pearson correlation test between the decreased pulse rate and comfort level are presented in Table 3. Decreased pulse rate and pressure comfort were positively correlated (*r* = 0.282**, *p* < 0.01), whereas touch pressure and mobility were not (*r* = 0.160, *p* > 0.05; and *r* = 0.121, *p* > 0.05, respectively). Compared to the overall score of the three aspects, the decreased pulse rate had a positive correlation (*r* = 0.201*, *p* < 0.05).

**Table 3:** Correlation of decreased pulse rate with comfort.

Variable	Pressure comfort	Touch comfort	Mobility	Overall score
Decreased Pulse Rate				
Pearson correlation	0.282^a^	0.160	0.121	0.201^b^
Sig. (2-tailed)	0.001	0.061	0.159	0.018
N (46 x 3 session)	138	138	138	138

a Correlation is significant at the 0.01 level (2-tailed).

b Correlation is significant at the 0.05 level (2-tailed).

## 4 Discussions

### 4.1 Main findings

#### 4.1.1 Decreased pulse rate as parasympathetic activity

After being subjected to the inflatable vest for 5 min, the user experienced a decrease in pulse rate, indicating an increase in parasympathetic activity. The effectiveness of deep pressure administration decreased in the second and third session. The inflatable vest is considerably comfortable, with no limitations to the user’s mobility; therefore, it can be categorized as a suitable therapeutic modality.

These findings align with Reynold’s research on the inflatable Vayu Vest, which showed a positive link between deep pressure and physiological responses. Even a 3-min intervention reduced arousal and anxiety and increased parasympathetic activity, as indicated by a significant increase in Respiratory Sinus Arrhythmia (RSA) [18]. In contrast, Chen’s study using Weighted Blankets found no significant difference in heart rate between the control and treatment groups, suggesting less therapeutic impact compared with Vayu Vest [19]. This highlights the potential superiority of the Inflatable Vest in providing therapeutic benefits over other deep-pressure tools, such as Weighted Blankets.

Deep pressure exerts a calming effect on the user through a decrease in sympathetic activity and an increase in parasympathetic activity, which can be accurately assessed using the peripheral pulse response. Khanade [16] reported that compared with non-traumatized individuals and those who did not have an autism spectrum disorder (ASD), those with individual trauma exhibited an accelerated heart rate when images related to their trauma were shown. This conforms with a study by Guinot Jimeno [20] who used heart rate indicators for changes in emotional activation and worry. A positive correlation was found in this study between scores on the Children’s Manifest Anxiety Scale (CMAS) and heart rate indicators.

#### 4.1.2 The parasympathetic mechanisms of calming regulation

The processing of touch stimuli involves the dorsal column-medial lemniscus (DCLM) pathway, which transmits somatosensory inputs from the skin to the somatosensory cortex via the thalamus. Furthermore, collateral networks allow these stimuli to reach other brain regions, such as the hypothalamus for autonomic and hormonal responses, the periaqueductal grey for opioid modulation, and the brainstem for autonomic regulation [21].

The autonomic nervous system (ANS) is essential for emotions, motivation, and involuntary bodily functions, such as heart rate and breathing [22]. It consists of two divisions: sympathetic and parasympathetic. The sympathetic system, known for “fight or flight”, response triggers arousal, while the parasympathetic, known as “rest and relax”, promotes relaxation [23]. Humans require flexible ANS modulation to adapt to social and cognitive challenges [24]. ANS responses are used to understand emotions, social situations, and stress [24,25].

#### 4.1.3 Subjective comfort aspect

Evaluating the user experience with a deep-pressure inflatable vest is crucial for assessing its effectiveness as a therapeutic tool. Comfort plays a significant role in patient compliance and well-being during therapy. In a study by Foo et al. (2018) that assessed subjective comfort with an active SMA compression garment, six of eight participants rated the vest as comfortable, with only one noting a fit issue [9]. However, the study overlooked user mobility. Our study addressed mobility concerns, with most participants reporting no compromise, allowing joint movement, and preserving their breathing capabilities.

### 4.2 Strengths of the study

This study investigated the therapeutic effectiveness of an inflatable deep-pressure vest, incorporating both physiological responses and subjective comfort rates, a novel approach in the field to explore the potential of inflatable vests to induce a calming effect. By incorporating a decrease in peripheral pulse rate and a high comfort level following vest administration, this study underscores its relevance in conditions such as anxiety and trauma-related disorders. Methodologically, this study integrates objective measures considering user experience and mobility, enhancing practical applicability and reliability. Overall, this study offers valuable insights into the therapeutic benefits of inflatable vests, contributing to a nuanced understanding of their mechanisms and potential as therapeutic tools.

### 4.3 Limitations of the study

This study had several limitations. First, the sample size was too small for generalizability. Second, focusing solely on adolescents aged 17–20 limits their relevance to other age groups. Additionally, using a one-time research design for comfort measurements and a one-group pretest-posttest design for physiological measurements without a control group may affect validity, requiring further research. Moreover, the short intervention duration of 5 min may not capture long-term effects or safety implications. Finally, the study’s exclusive focus on Indonesian individuals may limit its applicability to other cultural and racial groups since they may have different emotion regulation skills associated with physiological responses.

To enhance validity and generalizability, future research should use larger, more diverse samples across various age groups and cultural backgrounds. Implementing control groups and conducting longitudinal studies with extended intervention durations will provide clearer insights into long-term effects and safety. Additionally, employing repeated measurements and randomized controlled trials (RCTs) will strengthen evidence of the intervention’s effectiveness.

## 5 Conclusion

The inflatable deep pressure vest is comfortable and provides a calming effect; therefore, it is a prominent initial step for developing a new type of deep pressure modality for therapeutic devices, with new features including lightweight, handiness, and fashionability compared to previous forms. This deep pressure modality is called the autism hug machine portable seat/AHMPS and has been proven to have therapeutic effects on children with autism spectrum disorders.

## Additional information

No additional information is available for this paper.

## Funding

This study was supported by the 10.13039/501100005844Universitas Diponegoro under the scheme of International Research Publication No. 233-14/UN7.6.1/10.13039/501100004239PP/2020 and World Class Research No. 357-10/UN7.D2/10.13039/501100004239PP/10.13039/501100000026IV/2024.

## Institutional review board statement

The study procedures were approved by the Health Research Ethics Commission, Faculty of Medicine, Universitas Diponegoro, Semarang (No. 373/EC/KEPK/FK-UNDIP/IX/2021).

## Informed consent statement

The participants signed a consent form prior to the study.

## Data availability statement

The datasets used and/or analyzed in the present study are available from the corresponding author upon reasonable request.

## Declaration of Interest's statement

The authors declare no conflict of interest.

## Author agreement statement

We the undersigned declare that this manuscript is original, has not been published before and is not currently being considered for publication elsewhere. We confirm that the manuscript has been read and approved by all named authors and that there are no other persons who satisfied the criteria for authorship but are not listed. We further confirm that the order of authors listed in the manuscript has been approved by all of us. We understand that the Corresponding Author is the sole contact for the Editorial process. He/she is responsible for communicating with the other authors about progress, submissions of revisions and final approval of proofs.

## CRediT authorship contribution statement

**Mohamad Izzur Maula:** Writing – review & editing, Writing – original draft, Visualization, Validation, Supervision, Software, Resources, Project administration, Methodology, Investigation, Funding acquisition, Formal analysis, Data curation, Conceptualization. **Muhammad Imam Ammarullah:** Writing – review & editing, Writing – original draft, Visualization, Validation, Supervision, Software, Resources, Project administration, Methodology, Investigation, Funding acquisition, Formal analysis, Data curation, Conceptualization. **Hanifa Nur Fadhila:** Writing – review & editing, Writing – original draft, Visualization, Validation, Supervision, Software, Resources, Project administration, Methodology, Investigation, Funding acquisition, Formal analysis, Data curation, Conceptualization. **Ilham Yustar Afif:** Writing – review & editing, Writing – original draft, Visualization, Validation, Supervision, Software, Resources, Project administration, Methodology, Investigation, Funding acquisition, Formal analysis, Data curation, Conceptualization. **Hardian Hardian:** Writing – review & editing, Writing – original draft, Visualization, Validation, Supervision, Software, Resources, Project administration, Methodology, Investigation, Funding acquisition, Formal analysis, Data curation, Conceptualization. **Jamari Jamari:** Writing – review & editing, Writing – original draft, Visualization, Validation, Supervision, Software, Resources, Project administration, Methodology, Investigation, Funding acquisition, Formal analysis, Data curation, Conceptualization. **Tri Indah Winarni:** Writing – review & editing, Writing – original draft, Visualization, Validation, Supervision, Software, Resources, Project administration, Methodology, Investigation, Funding acquisition, Formal analysis, Data curation, Conceptualization.

## Declaration of competing interest

The authors declare that they have no known competing financial interests or personal relationships that could have appeared to influence the work reported in this paper.
